# iTRAQ-based quantitative proteomic analysis of heat stress-induced mechanisms in pepper seedlings

**DOI:** 10.7717/peerj.11509

**Published:** 2021-06-03

**Authors:** Jing Wang, Chengliang Liang, Sha Yang, Jingshuang Song, Xuefeng Li, Xiongze Dai, Fei Wang, Niran Juntawong, Fangjun Tan, Xilu Zhang, Chunhai Jiao, Xuexiao Zou, Wenchao Chen

**Affiliations:** 1 Vegetable Research Institute, Hunan Academy of Agricultural Sciences, Changsha, China; 2 Longping Branch, Graduate School of Hunan University, Changsha, China; 3 College of Horticulture and Landscape, Hunan Agricultural University, Changsha, China; 4 Hubei Academy of Agricultural Sciences, Wuhan, China; 5 Faculty of Science, Department of Botany, Kasetsart University, Bangkok, Thailand

**Keywords:** Pepper, Heat stress, Physiological, Proteomics

## Abstract

**Background:**

As one of the most important vegetable crops, pepper has rich nutritional value and high economic value. Increasing heat stress due to the global warming has a negative impact on the growth and yield of pepper.

**Methods:**

To understand the heat stress response mechanism of pepper, an iTRAQ-based quantitative proteomic analysis was employed to identify possible heat-responsive proteins and metabolic pathways in 17CL30 and 05S180 pepper seedlings under heat stress.

**Result:**

In the present study, we investigated the changes of phenotype, physiology, and proteome in heat-tolerant (17CL30) and heat-sensitive (05S180) pepper cultivars in response to heat stress. Phenotypic and physiological changes showed that 17CL30 had a stronger ability to resist heat stress compared with 05S180. In proteomic analysis, a total of 3,874 proteins were identified, and 1,591 proteins were considered to participate in the process of heat stress response. According to bioinformatic analysis of heat-responsive proteins, the heat tolerance of 17CL30 might be related to a higher ROS scavenging, photosynthesis, signal transduction, carbohydrate metabolism, and stress defense, compared with 05S180.

## Introduction

Pepper (*Capsicum annuum* L.) is one of the crops with the largest cultivated area in China, which greatly contributes to the vegetable supply and farmers’ income. Capsaicin and capsanthin extracted from pepper or its other components, such as vitamin and trace element, can improve people’s appetite and health. It is well known that pepper can well grow in warm place but sensitive to high temperature. Pepper will suffer from heat stress when the ambient temperature exceeds 32 °C (Guo et al., 2014), and its yield and quality are seriously reduced by the high temperature during the hot summer. Therefore, it is necessary to investigate the molecular mechanism underlying the pepper’s response to heat stress in order to provide crucial information for the research on complex traits and breeding heat-tolerant pepper cultivars.

Heat stress is one of the adverse environment factors affecting the growth and productivity of plants, resulting in wilting or even death of plants (Lesk, Rowhani & Ramankutty, 2016; Wahid et al., 2007). According to the predication of IPCC (Intergovernmental Panel on Climate Change), the global temperature is likely to increase by about 2−5 °C by the end of 21th century (https://www.ipcc.ch/). In hot summer, the temperature in some parts of China usually goes above 40 °C, especially in the south of the Yangtze River (http://data.cma.cn/). According to the statistics, the pepper yield will be reduced by about 70%, leading to serious economic loss (Gajanayake et al., 2011).

Until now, many studies have investigated the molecular mechanism of heat stress responses. To cope with heat stress, a set of defense mechanisms are activated to protect plants from the heat stress-induced damage, including the change of signal transduction and transcriptional regulation, the accumulation of osmolytes, the production of reactive oxygen species (ROS) scavenging enzymes, and the synthesis and accumulation of heat shock proteins (HSPs) (Ding, Shi & Yang, 2020; Gong et al., 2020; Ohama et al., 2017; Vu, Gevaert & De Smet, 2019; Wahid et al., 2007). The generation and accumulation of ROS caused by heat stress can lead to the peroxidation of membrane lipids and proteins as well as carbohydrate oxidation and DNA damage (Gill & Tuteja, 2010; Zhang et al., 2019). To scavenge and detoxify ROS, plants have evolved both enzyme and non-enzyme systems (Gill & Tuteja, 2010). The non-enzymatic antioxidants, including glutathione (GSH) and ascorbate, and enzymatic antioxidants, such as peroxidase (POD), glutathione S-transferase (GST), ascorbate peroxidase (APX), dehydroascorbate reductase (DHAR), catalase (CAT), and superoxide dismutase (SOD), play critical roles in the detoxification of ROS (Shi et al., 2017; Smirnoff, 2003; Wang et al., 2020b). The ROS scavenging capacity of plants may be related to their tolerance to heat stress. Furthermore, HSPs, as molecular chaperones, are involved in folding, transferring and assembly of proteins, and they also can repair or remove denatured proteins in plant cell. HSP90, HSP70 and small HSPs (sHSPs) have been demonstrated to protect plants against the heat stress (Wang et al., 2020a; Wang et al., 2004).

In recent years, quantitative proteomic analysis has been applied to investigate the response to abiotic stress in plants (Cao et al., 2017; Tian et al., 2015; Zhan et al., 2019). Proteomic analysis of plants under heat stress has been widely performed, and some heat-responsive proteins and metabolic pathways have been identified to play important roles in the process of plant response to heat stress (Han et al., 2009; Lu et al., 2017). Under heat stress, proteins involved in the antioxidative progress, photosynthesis and HSP-related protection mechanisms are induced in rice (Han et al., 2009). Proteins, such as chaperones, GSTs, and thioredoxin (Trx), are induced when wheat is suffered from heat stress (Lu et al., 2017). It is meaningful to perform proteomic analyses to further explore the molecular mechanisms involved in heat tolerance of pepper. In the present study, heat-tolerant (HT) and heat-sensitive (HS) pepper seedlings were subjected to heat stress (40 °C) to identify the differentially accumulated proteins (DAPs) in different pepper genotypes, and the contents of proteins in pepper leaves collected at four time points were identified and quantified by using the iTRAQ-based quantitative proteomic approach. Moreover, we aimed to identify some key proteins and pathways related to heat stress response in pepper. In addition, we also attempted to provide a new insight into the molecular mechanisms involved in heat tolerance of pepper.

## Materials & Methods

### Plant materials and heat treatment

Two pepper cultivars 17CL30 (HT) and 05S180 (HS) were obtained from Vegetable Institution of Hunan Academy of Agricultural Science. The seed germination and seedling management of pepper were carried out as previously described (Wang et al., 2019). Six-leaf stage seedlings of HT and HS cultivars were subjected to heat stress at 40 °C, and the leaves were harvested at 0, 3, 28 and 48 h for physiological assays and proteomic analysis. Three biological replicates were performed for each treatment.

### Measurement of physiological indices

Control (0 h) and 48-h heat-treated samples were used for the measurement of physiological indices. The relative water content (RWC) of leaves was determined as previously described (Zegaoui et al., 2017). The contents of proline, soluble sugar, malondialdehyde (MDA), reduced GSH, POD, GST, SOD and CAT were determined using commercially available kits (Nanjing Jiancheng Bioengineering Institute, China) according to the manufacturer’s instructions.

### Protein extraction, iTRAQ labeling, and data analysis

Total proteins were extracted from 24 samples using plant total protein extraction kit (PP0601-100; Beijing Bio-Fly Bioscience Co., Ltd., China) according to the manufacturer’s instructions. The iTRAQ labeling and analysis were performed at Biomarker (Beijing). The concentrations of the extracts were determined using the Bradford method. The protein extracts (200 µg each) were reduced by 1M DTT (5 µL), alkylated by 1M (20 µL) IAA, and then digested by the FASP (filtered aide sample preparation) method using trypsin V5113 (Promega, CA, USA). Peptides (100 µg) were labelled with iTRAQ Reagent-8Plex Multiplex Kit (AB Sciex, MA, USA) according the manufacturer’s instructions. HT0 h, HT3 h, HT28 h, HT48 h, HS0 h, HS3 h, HS28 h, and HS48 h were labelled with 113, 114, 115, 116, 117, 118, 119, and 121 tags respectively. Easy nLC/ Ultimate 3000 (Thermo Scientific, CA, USA) was used to carry out High pH RP chromatography. Solvent A (98% H_2_O, pH10) and solvent B (98% ACN, pH10) were used to perform HPLC. The labeled peptide mixtures were reconstituted in solvent A and were loaded onto a 150 µm ×120 mm C18 column containing 1.9 µm particles (Thermo Scientific, CA, USA). The gradients were as follows: 0-3 min, 3% B; 3−5.1 min, 0% B; 5.1–10 min, 5% B; 10–35 min, 18% B; 35–40 min, 34% B; 40–53 min 95% B; 53–58 95% B. Samples were collected for 5–58 min and eluents were collected every 1.5 min. Then, the peptide fractions were freeze-dried. The dried samples were reconstituted in formic acid (5 µL 0.5%) and loaded on a C18 nanoLC trap column (1.9 µm, 150 µm ×120 mm), and then were washed using Nano-RPLC Buffer A (0.1% FA, 2% ACN) at 300 nL/min for 10 min. The peptide fractions were analyzed using Q Exactive HF-X (Thermo Scientific, CA, USA). The analysis time was 90 min and the detection method was positive ion. The spray voltage was set as 2.5 KV and ion transfer tube temperature was 150 °C. MS data was acquired using a data-dependent top10 method, which dynamically selected the most abundance precursor ions from the survey scan (350–1, 800 m/z) for HCD (higher energy collisional dissociation) fragmentation. Automatic gain control (AGC) target was set to 3 ×106, maximum inject time to 20 ms, and dynamic exclusion duration to 30 s. Survey scans were acquired at a resolution of 60,000 at m/z 200 and resolution for HCD spectra was set to 15,000 at m/z 200, AGC target was set to 3 × 105, a maximum inject time to 45 ms, and isolation width was 2 m/z. The raw file was processed using the Mascot engine embedded into the Proteome Discoverer 2.1 against the pepper protein database (Zunla-1 version 2). The principal component analysis (PCA) was used to evaluate the relationship among 24 samples. *T*-test was used to compare the differential expressions of proteins. Proteins with fold-changes >1.2 or <0.83 and *P*-value <0.05 were considered as DAPs.

### Bioinformatic analysis

The functional classification of DAPs was performed by the Clusters of Orthologous Groups (COGs) protein database (https://www.ncbi.nlm.nih.gov/COG/). Gene Ontology (GO) database (http://geneontology.org/) was used for functional annotation of DAPs. The prediction of metabolic pathways was carried out with Kyoto Encyclopedia of Genes and Genomes (KEGG) database (https://www.genome.jp/kegg).

## Results

### Phenotype and physiological responses of HT and HS under heat stress

**Figure 1:**
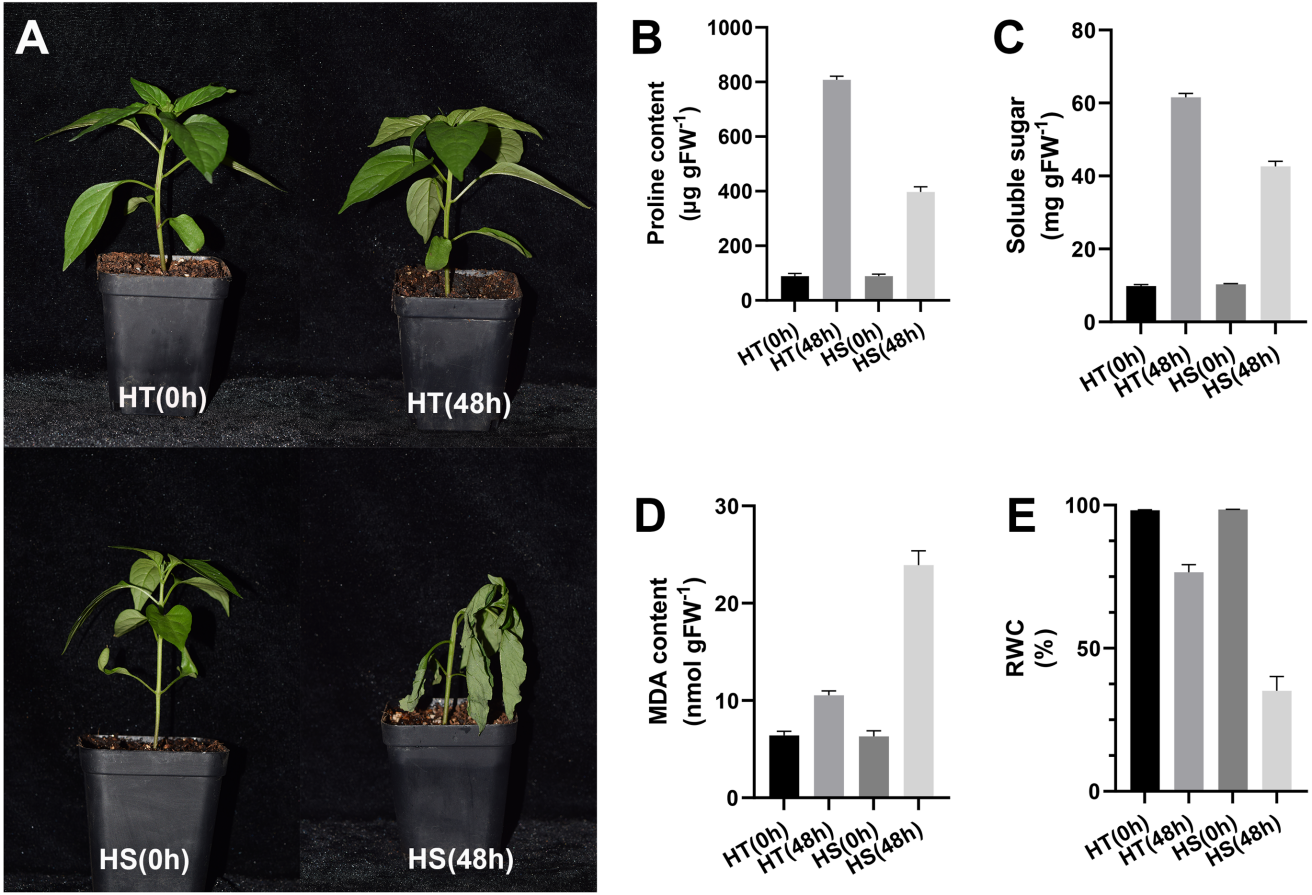
Phenotype (A) and physiology responses of heat-tolerant and heat-sensitive pepper genotypes including proline (B), soluble sugar (C) MDA (D) and RWC (E) under 0 and 48 h heat stress. The error bars represent SD.

After 48 h of heat stress, HS seedlings showed more wilting than HT, suggesting that the effect of heat stress on pepper seedlings was slighter in HT compared with HS (Fig. 1A). After 48 h of heat stress, the contents of proline and soluble sugar were increased in two pepper genotypes, while their contents in the HT genotype were higher compared with the HS genotype (Figs. 1B, 1C). Compared with the control samples, the content of MDA was significantly increased both in HT and HS after 48 h of heat stress. However, HS showed a stronger up-regulation (Fig. 1D). RWC values after 48 h of heat stress were decreased in leaves, and such reduction was greater in HS compared with HT (Fig. 1E).

### iTRAQ data analysis and responses of DAPs to heat stress

**Figure 2:**
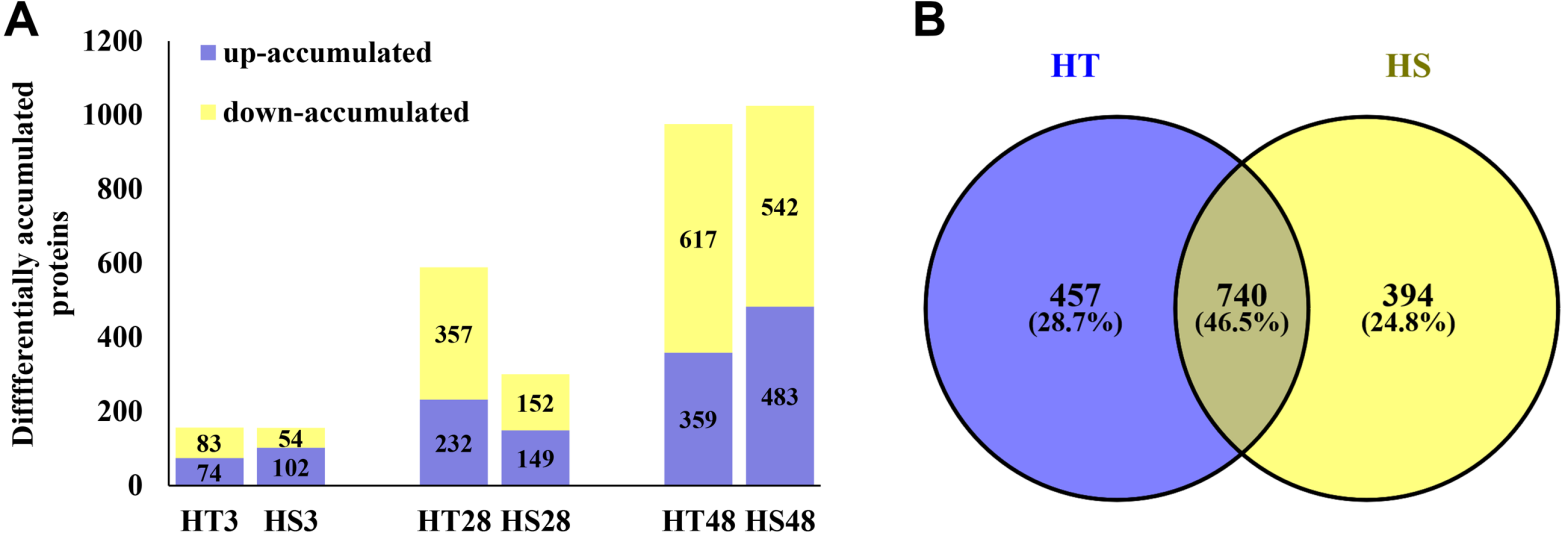
The differentially accumulated proteins (DAPs) in HT and HS genotypes under heat stress. (A) The number of the DAPs identified in HT and HS at different time point of heat stress. (B) Veen graph of the DAPs of HT and HS.

Based on the iTRAQ experiment, a total of 1,628,623 spectra were identified from pepper seedlings. Moreover, 23,706 peptides, 21,335 unique peptides, and 5,098 proteins were detected from 193,696 known spectra (Fig. S1A). The MS data were deposited into the identifier PXD019556. Fig. S1B shows the mass distribution of proteins, and the majority of them were 10–70 kDa in size. In addition, sequence coverage of most proteins was <35% (Fig. S1C). The coefficient of variation of replicates was calculated to evaluate the reliability of proteomic data. The result showed that proteins with 20% coefficient of variation were more than 80% of identified proteins (Fig. 1D), suggesting that the data were reliable. In a PCA model based on 24 samples, control (0 h) and heat-treatment samples (3, 28 and 48 h) were clearly separated (Fig. S2). PCA1 accounted for 40.30% of the variability, while the PCA2 accounted for 38.30% of the variability (Fig. S2). All identified proteins were listed in Table S1. Compared with the control samples, a total of 1,591 DAPs were identified under heat stress in HT and HS. Among these DAPs, 157/156, 589/301, and 976/1,025 were significantly accumulated in HT/HS under heat stress at 3, 28, and 48 h, respectively (Fig. 2A). In short, there were more DAPs identified in HT compared with HS (Fig. 2A). Moreover, 457 and 394 specific DAPs were identified in HT and HS, respectively, while 740 common DAPs were found both in HT and HS (Fig. 2B).

### Functional analysis of DAPs in response to heat stress

**Figure 3:**
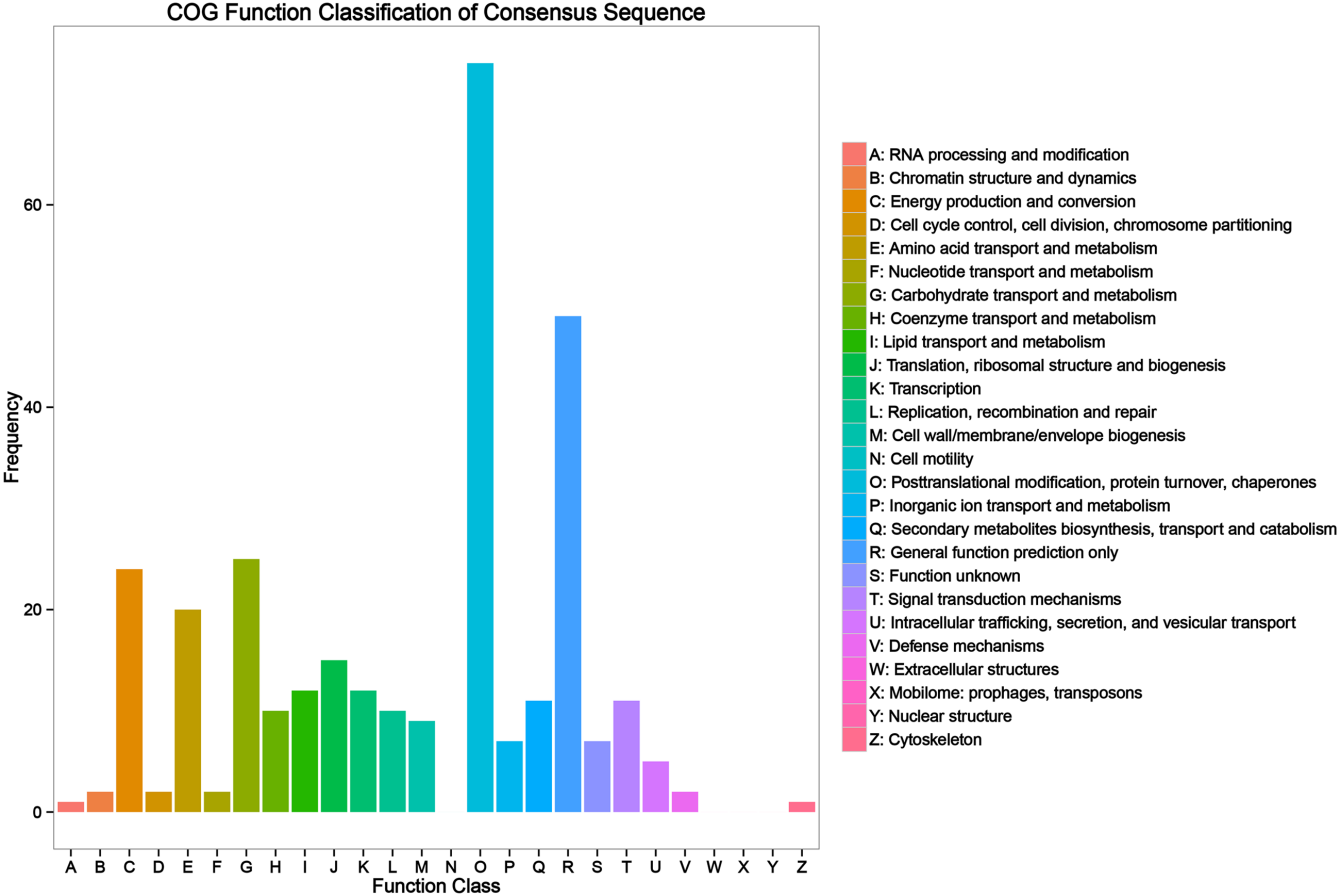
Clusters of Orthologous Groups (COG) annotation analysis of all heat responsive proteins in HT and HS.

Of the 1,591 DAPs, 997 DAPs were categorized into 24 categories using COGs database. The largest group was general function prediction only, followed by post-translational modification, protein turnover, chaperones, translation, ribosomal structure and biogenesis, energy production and conversion, carbohydrate transport and metabolism, amino acid transport and metabolism, signal transduction mechanism, and defense mechanism (Fig. 3A and Table S2).

**Figure 4:**
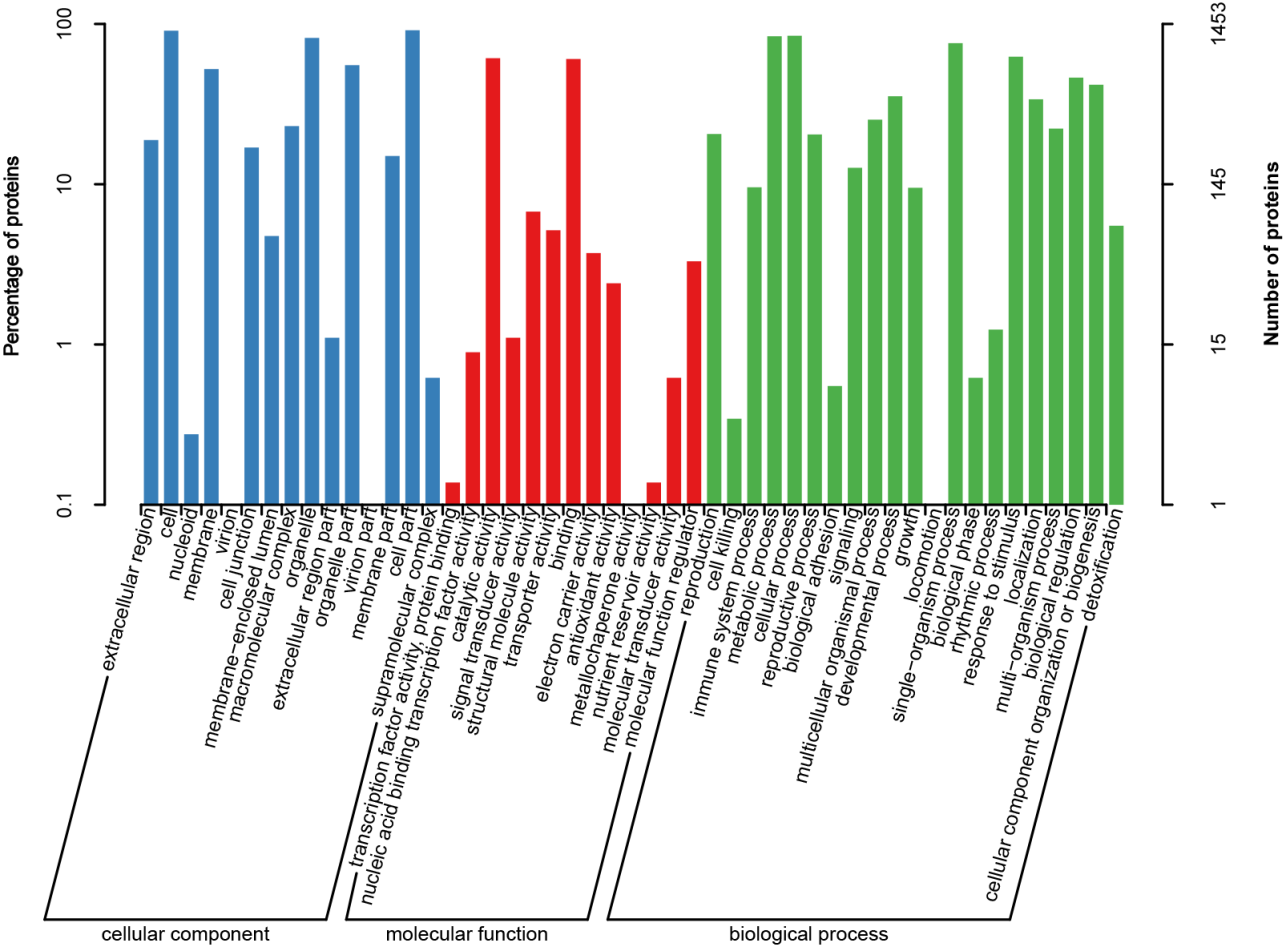
Gene Ontology (GO) annotation of all heat responsive proteins in HT and HS.

To further characterize all heat-responsive proteins, these DAPs identified in HT and HS were subjected to GO analysis. Of the 1,591 DAPs identified in HT and HS under heat stress, 1,453 proteins were annotated into three groups as cellular component (CC), molecular function (MF), and biological process (BP). GO analysis showed that cell part, cell, and organelle were the major CC terms; catalytic activity, binding, structural molecule activity and transporter activity were the dominant MF terms; and cellular process, signaling, metabolic process, single-organism process and response to stimulus were the most dominant BP terms (Fig. 4).

**Figure 5:**
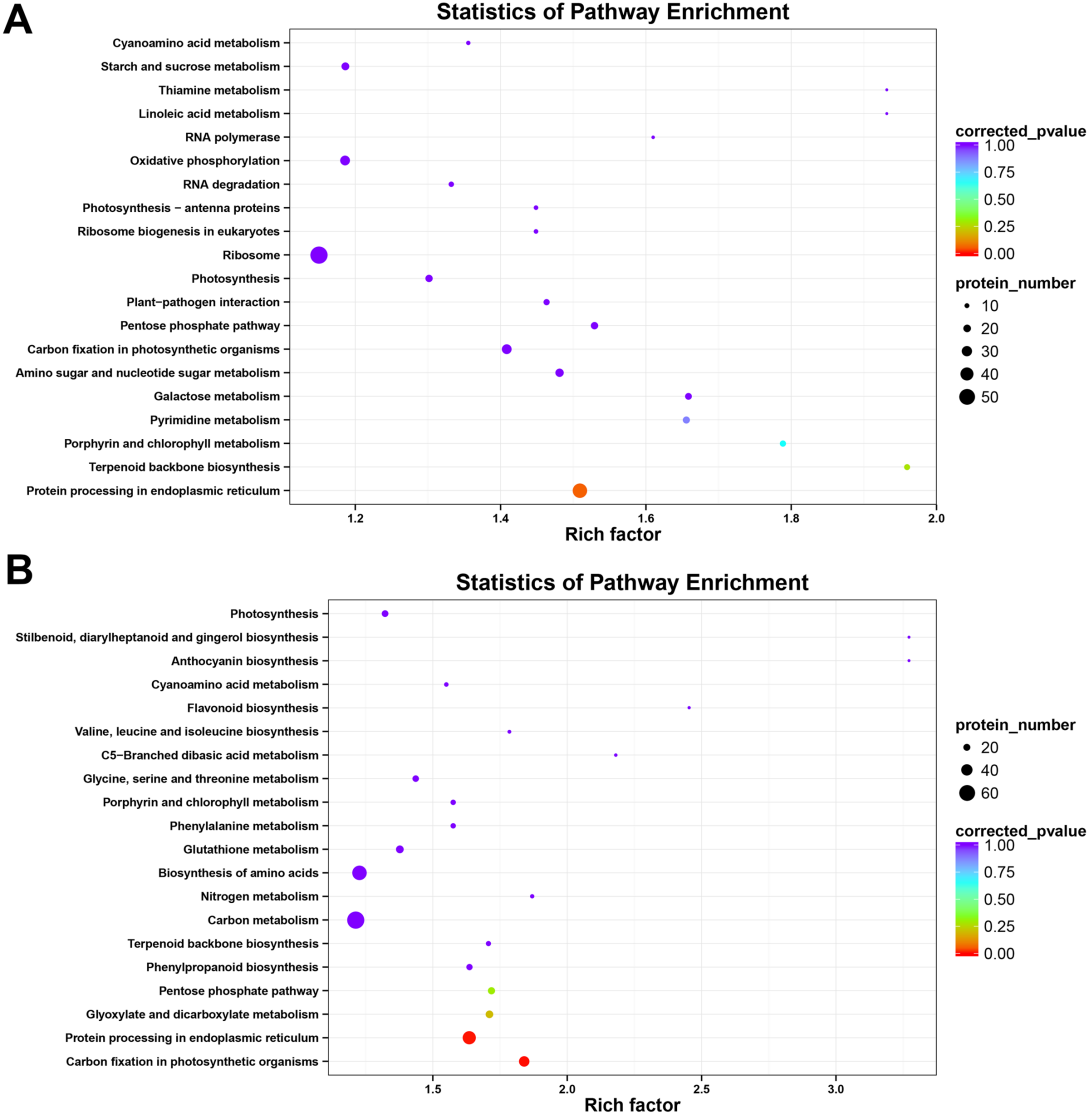
Kyoto Encyclopedia of Genes and Genomes (KEGG) pathway enrichment of DAPs in HT (A) and HS (B).

To further investigate the major metabolic pathways responding to heat stress, KEGG enrichment analysis was carried out with heat-responsive proteins from HT and HS. The result showed that DAPs were matched to 99 KEGG pathways in HT and 106 KEGG pathways in HS. Heat stress could affect protein processing in photosynthesis, endoplasmic reticulum, carbon fixation in photosynthetic organisms, and pentose phosphate pathways in both two genotypes (Fig. 5). Furthermore, KEGG metabolic pathways, including pyrimidine metabolism, galactose metabolism and amino sugar and nucleotide sugar metabolism, were highly enriched in HT, whereas other pathways, such as phenylpropanoid biosynthesis, carbon metabolism, nitrogen metabolism, biosynthesis of amino acids, glutathione metabolism, phenylalanine metabolism, and glycine, serine and threonine metabolism, were considerably enriched in HS (*P* < 0.05, Tables S3 and S4).

### Antioxidants in response to heat stress

**Figure 6:**
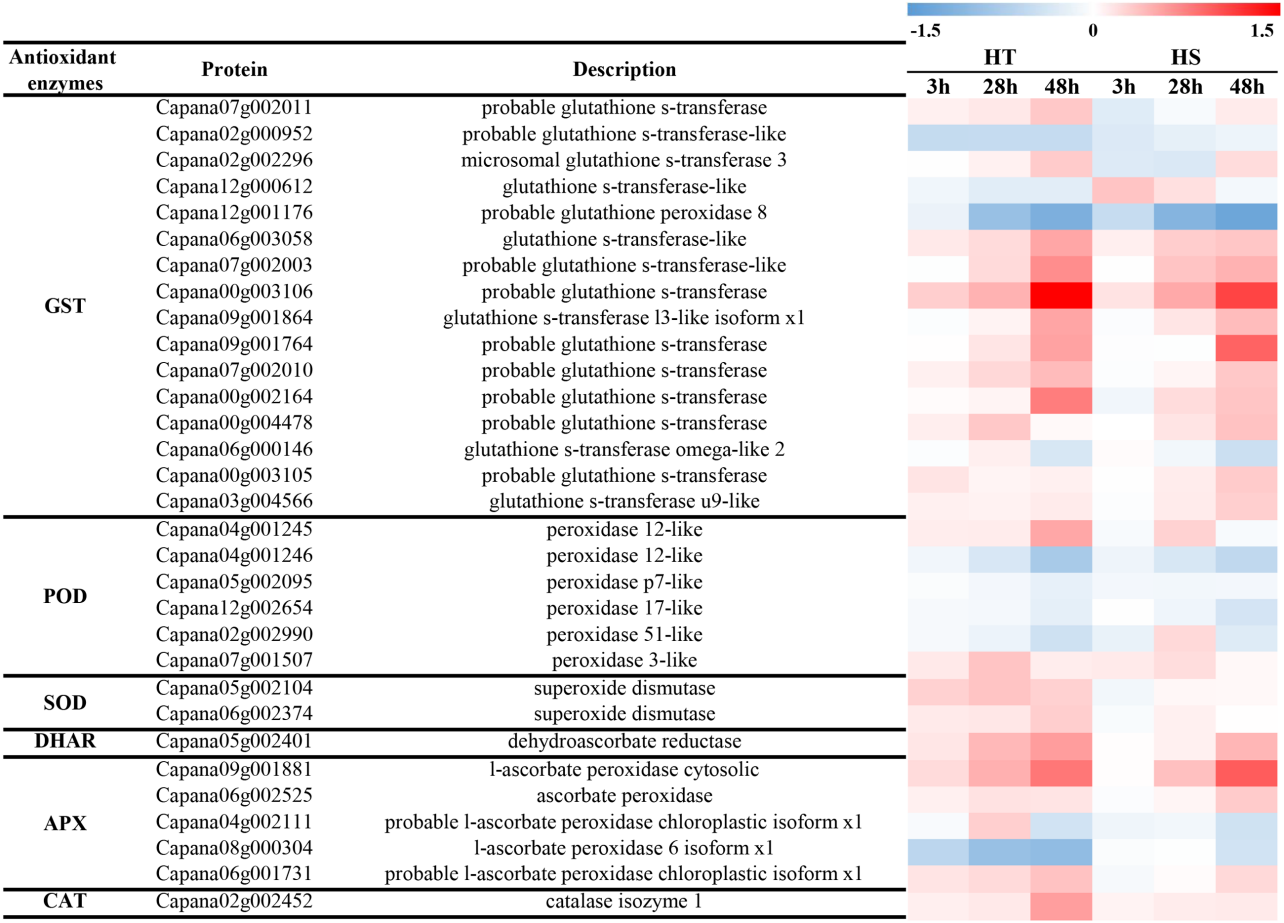
The change of enzymatic antioxidants of HT and HS in response to heat stress based on the proteomics data.

In response to heat stress, antioxidant enzymes (GST, POD, SOD, DHAR, APX and CAT) were differentially accumulated according to proteomic data. Under heat stress, most GSTs were significantly increased with the increase of treatment time (Fig. 6A). Some of them were accumulated in both two genotypes, while two of them were suppressed in response to heat stress (Capana02g000952, Capana12g001176). Among six identified PODs, POD 12-like and 3-like were significantly increased in HT, while POD p7-like, 17-lke, and 51-like were decreased in HT and HS (Fig. 6A). Under heat stress, two SODs were identified in response to heat stress, and both of them were greatly increased in HT, while there were no obvious changes in HS (Fig. 6A). One DHAR and one CAT were significantly increased in HT, while they were slightly increased in HS (Fig. 6A). In addition, most APXs were increased in HT, while they were only three APXs (Capana09g001881, Capana06g002525, Capana06g001731) were increased in HS.

**Figure 7:**
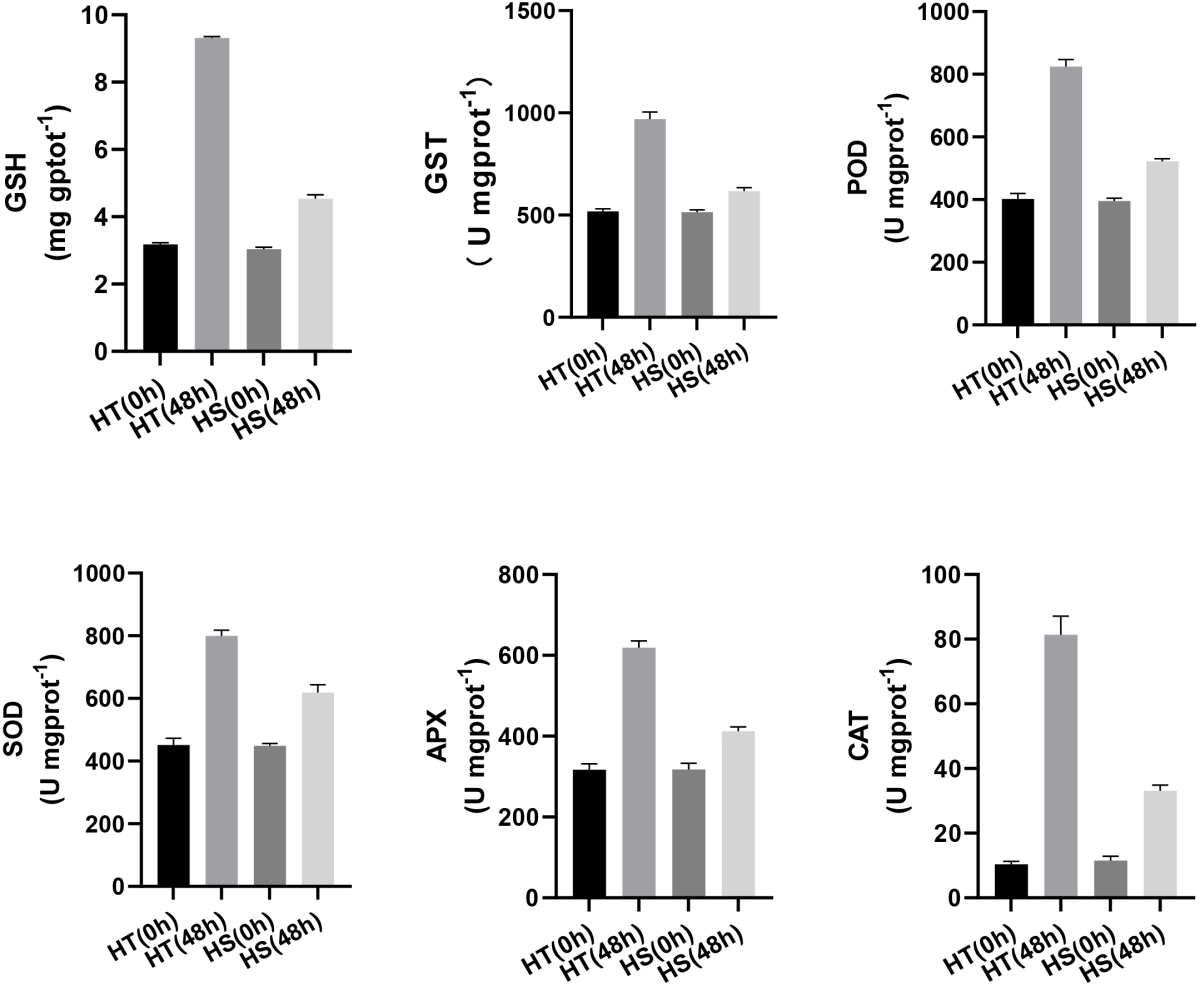
The content of GSH (A) and the activities of GST (B), POD (C), SOD (D), APX(E), and CAT(F) in HT and HS under 0, and 48 h heat stress using commercially available kits. The error bars represent SD.

Furthermore, to further compare the differences of antioxidant capacity between HT and HS, the content of GSH and the activities of GST, POD, SOD, APX, and CAT were determined at 0 and 48 h after the heat treatment. Figure 7A shows that the content of GSH was increased in both two genotypes under heat stress. However, its content was higher in HT compared with HS. The activities of GST and POD were higher in HT compared with HS after 48 h of heat stress (Figs. 7B, 7C). Compared with the control samples, the activities of SOD, APX, and CAT were significantly increased in HT. However, there were no obvious changes in HS (Figs. 7D, 7E, 7F).

### Specific DAPs of HT in response to heat stress

To further investigate the mechanism underlying the higher heat tolerance in HT, the functions of 457 specific DAPs were studied based on GO analysis (Table S5). In short, these DAPs were mainly enriched in protein autophosphorylation, hexokinase-dependent signaling, and regulation of ROS metabolic process. Among these proteins, 95 proteins were up-regulated at each treatment time point under heat stress, including glutamate decarboxylase (Capana00g003786), 26s protease regulatory subunit 8 homolog a (Capana01g002147), sHSP (18.2 kDa class I heat shock protein), universal stress protein a-like protein, and so on (Table S5). Moreover, some DAPs related to signal transduction were also identified in HT, such as calcium-dependent protein kinase (Capana04g002552), serine threonine-protein kinase ht1 (Capana07g001488), and atp-dependent 6-phosphofructokinase 6 (Capana07g001111).

## Discussion

Heat stress seriously restricts the quality and yield of pepper. Therefore, it is necessary to uncover the mechanisms underlying the heat stress response of pepper. In the present study, we investigated the phenotypic and physiological changes of two pepper seedlings under heat stress. Furthermore, the comparative analysis of heat-induced proteomic changes was carried out to obtain a global view of heat stress responses in pepper. As a result, we identified 1,591 heat-responsive proteins in pepper leaves, and the functions of these proteins were annotated using public databases. The functions of these proteins and main related metabolic processes were further discussed below.

### DAPs involved in signal transduction

When plants are subjected to abiotic stress, the complex signal networks are regulated to cope with the unavoidable environmental conditions and keep growth and development (Abreu et al., 2013; Kaur & Gupta, 2005). It is well known that protein kinases are involved in signal transduction by mediating the response of plant cell to external environment (Abreu et al., 2013). Overexpression of rice serine-threonine protein kinase SAPK4 resulted in improved the ability of salt stress acclimation (Diedhiou et al., 2008). Receptor-like protein kinases are also believed to play an important role in abiotic stress (Jung et al., 2015), and overexpression of receptor-like kinase ER in rice and tomato improves thermotolerance (Shen et al., 2015). In the current research, four serine-threonine protein kinases and one receptor-like protein kinase were increased in HT compared with HS (Table S6). Meanwhile, calmodulin plays a critical role in heat shock signal transduction, and the expressions of calmodulin genes are up-regulated after heat treatment (Al-Quraan, Locy & Singh, 2010). Herein, calmodulin and calmodulin-7 were up-regulated in HT and HS (Table S6). These findings suggest that protein kinase and calmodulin functioned as positive regulators to improve the heat tolerance of HT seedlings. In addition, 14-3-3 proteins were also identified as cold/salt/drought-responsive proteins, respectively (Abreu et al., 2013; Yang et al., 2019; Zhang et al., 2015). 14-3-3 protein 4, 14-3-3 protein 9, and 14-3-3 protein 10 were more accumulated in HT compared with HS under heat stress. This finding indicated that 14-3-3 proteins executed an important function in resistance to heat stress in pepper seedlings. The more accumulation of these proteins involving in signal transduction might contribute to the heat tolerance of HT seedlings.

### DAPs involved in photosynthesis

Photosynthesis is the most basic physiological process for plant growth and development (Ashraf & Harris, 2013; Lu et al., 2017). Owing to its complex molecular mechanisms and demand for multiple enzymes, photosynthesis is often cited as the most heat-sensitive physiological process (Centritto et al., 2011; Shi et al., 2017). Previous studies have found that the photosynthesis is inhibited by heat stress (Ashraf & Harris, 2013; Li et al., 2015). It is well documented that PSII is the most heat-sensitive component of photosynthesis (Ashraf & Harris, 2013). Our study suggested that eight proteins related to PSII were down-regulated, such as two PsbA, PsbB, PsbC, PsbD, PsbS, PsbR, and Psb28 (Table S6). Meanwhile, nine proteins (three PsaA, PsaB, two PsaE, PsaF, PsaH, PsaO) involved in PSI were differentially accumulated under heat stress (Table S6). Most of them were down-regulated under heat stress. Furthermore, DAPs related to cytochrome b6/f complex, such as PetB, PetA, PetH, PetC, and PetE, photosynthetic eletron transport including PetE and PetH, and F-type ATPase enzyme with multiple subunits (a, alpha, two b, beta, delta, epsilon and gamma), were also down-regulated. These finding indicated that heat stress could dramatically decrease PSII compared with PSI. Overall, the photosynthesis of HT and HS was both decreased under heat stress. However, HT could maintain relatively high photosynthesis compared with HS.

### DAPs involved in carbohydrate metabolism

Glycolysis, tricarboxylic acid (TCA) cycle, and pentose phosphate pathway are important components of carbohydrate metabolism. The glycolysis and TCA cycle are inhibited with the increase of temperature (Wang et al., 2020b). Table S6 shows that there were 41 proteins involved in glycolysis, and most of them were down-regulated after heat stress, such as glucose-6-phosphate isomerase, hexokinase, pyruvate dehydrogenase, and so on. Interestingly, HT could maintain a higher glycolysis under heat stress owing to the relatively high activities of related enzymes compared with HS. In addition, nine proteins were identified to be involved in TCA cycle. The TCA cycle can provide essential energy and precursors for the synthesis of substances, and it can also be affected by abiotic stress (Williams et al., 2010; Zhang et al., 2017). Pyruvate dehydrogenase complex (PDC) is a type of multienzyme complex located in the mitochondrial matrix. PDC, involved in the irreversible oxidation of pyruvate, plays an important role in energy metabolism of mitochondrial respiratory chain (Yu et al., 2012). Here, we found that the abundance of pyruvate dehydrogenase e1 component subunit beta (PDHB) was reduced in HT under heat stress, while only little change was found in HS (Table S6). This finding indicated that the activity of TCA cycle was more significantly decreased in HT compared with HS. It has been reported that the reduced consumption of organic acids caused by the decreased activity of TCA cycle may contribute to the synthesis of compounds to cope with stressful condition (Kiani-Pouya et al., 2017; Widodo et al., 2009). Therefore, reducing the activity of TCA cycle in HT might be conducive to the synthesis of osmosis substances to cope with heat stress. The pentose phosphate pathway (PPP) is also a major source of reductant and intermediates for substance biosynthesis (Kruger & Von Schaewen, 2003). In our present study, the key members of the PPP, including ribose-5-phosphate isomerase, ribulose-phosphate 3, ATP-dependent 6-phosphofructokinase, 6-phosphogluconate decarboxylating 3, and others, were reduced in both HT and HS (Table S6). However, the activities of related enzymes in HS were lower compared with HT, suggesting that HS suffered more from oxidative damage caused by heat stress.

### DAPs involved in stress defense

In the present study, 93 DAPs were identified to be involved in the process of heat stress defense, including enzymatic and non-enzymatic antioxidants, and molecular chaperones.

Heat stress can disturb the balance between generation and scavenging of ROS and resulting in damaged membrane system and other cellular components. To cope with the peroxidation damages, plants have developed an efficient enzymatic and non-enzymatic antioxidant system (Gill & Tuteja, 2010). It is well established that GSH is one of the important metabolites in plant cells and organelles, which functions as the intracellular defense against oxidative damage induced by ROS. In this study, compared with the corresponding control group, the content of GSH was increased in HT, while it was slightly increased in HS (Fig. 6B), suggesting that HT was subjected to minor damage from ROS. It has also been suggested that the up-regulation of antioxidant enzymes can regulate the ROS level and protect the plant cells from oxidative damage under heat stress (Das & Roychoudhury, 2014; Lu et al., 2017; Wang et al., 2020b; Xu et al., 2018). In the present study, some antioxidant enzymes were identified, including GST, POD, SOD, APX, and CAT (Fig. 6A). Moreover, we found that some TRXs were accumulated under heat stress, which can function in the process of plant cell against oxidative damage (Table S6) (Laloi et al., 2004; Vieira Dos Santos & Rey, 2006). Interestingly, HT showed a higher ability to adapt to high temperature with more accumulation of antioxidants compared with HS, which was consistent with the minor MDA content in HT compared with HS under heat stress.

Molecular chaperones are crucial proteins, which can prevent the synthesis of misfolded proteins and maintain the intracellular homeostasis in plant under suitable or stressful condition (Kang, Park & Kwak, 2013; Voth & Jakob, 2017; Wang et al., 2004). HSPs are one of chaperones, which are induced when plants suffer from heat stress (Merret et al., 2017). Over-expression of CaHSP70a contributes to the higher heat tolerance, and CaHSP70a protein is also accumulated in pepper under heat stress (Kim & Hwang, 2015). In addition, HSP90 (Guo et al., 2012) and sHSPs (Waters & Vierling, 2020) are also involved in the response of heat stress. In our present study, 10 HSP70s, five HSP90s, and 11 sHSPs were up-regulated after heat stress (Table S6). Previous studies indicated that ubiquitin-conjugating enzyme and other chaperone protein help plants cope with the adverse environment (Sharma & Bhatt, 2017). The up-regulated of these proteins might indicate that they functioned as the molecular chaperones and played a critical role in response to heat stress in pepper.

## Conclusions

**Figure 8:**
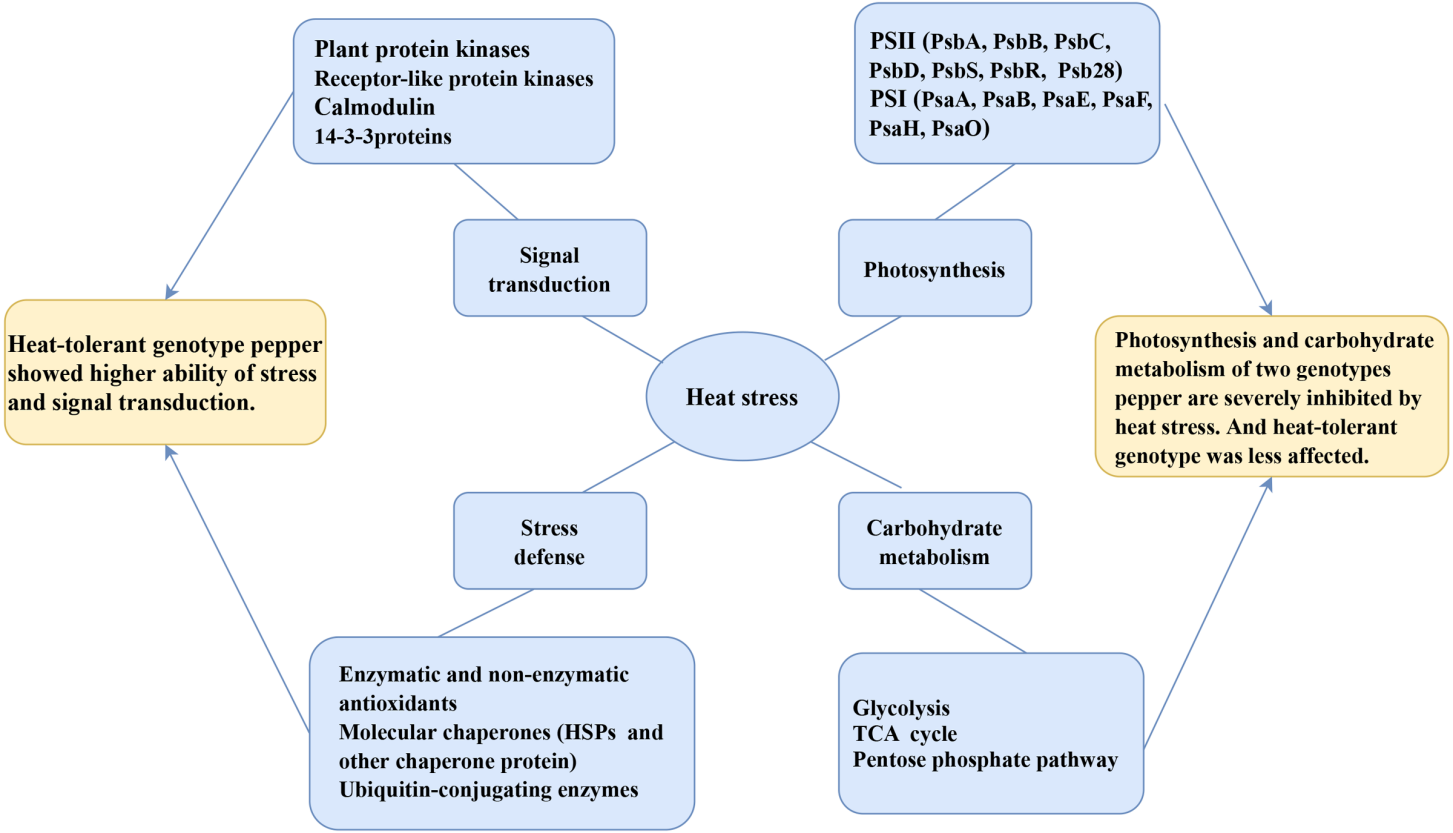
The model of pepper response to heat stress based on physiological and proteomic changes.

In the present study, we studied the physiological and proteomic changes of HT and HS seedlings to uncover the molecular mechanisms in heat tolerance. A total of 1,591 DAPs were identified as heat-responsive proteins in pepper seedlings. The bioinformatic analysis revealed that these DAPs were involved in energy production and conversion; carbohydrate transport and metabolism; catalytic activity; structural molecule activity and transporter activity; and response to stimulus. The KEGG enrichment analysis showed that photosynthesis, endoplasmic reticulum, terpenoid backbone biosynthesis, porphyrin and chlorophyll metabolism, carbon fixation in photosynthetic organisms, and pentose phosphate pathways were significantly related to the response to heat stress. Furthermore, some proteins were confirmed to play a critical role in protein folding, osmoregulation and ROS scavenging. A model of pepper response to heat stress was generated based on physiological and proteomic changes (Fig. 8). Collectively, our findings provided valuable insights into the molecular mechanisms underlying the heat tolerance of pepper and might be useful reference for the breeding of new pepper variety with heat resistance.

##  Supplemental Information

10.7717/peerj.11509/supp-1 Supplemental Information 1 A principal component analysis (PCA) of iTRAQ data

10.7717/peerj.11509/supp-2 Supplemental Information 2 Basic information statistic of iTRAQ-based proteome data. (A) Statistics of basic information of protein identification, (B) protein mass distribution, (C) distribution of protein sequence coverage, and (D) coefficient of variation of replicates.

10.7717/peerj.11509/supp-3 Supplemental Information 3 All proteins identified in HT and HS

10.7717/peerj.11509/supp-4 Supplemental Information 4 Clusters of Orthologous Groups (COG) annotation analysis of all heat responsive proteins in HT and HS

10.7717/peerj.11509/supp-5 Supplemental Information 5 Kyoto Encyclopedia of Genes and Genomes (KEGG) pathway enrichment of DAPs in HT

10.7717/peerj.11509/supp-6 Supplemental Information 6 Kyoto Encyclopedia of Genes and Genomes (KEGG) pathway enrichment of DAPs in HS

10.7717/peerj.11509/supp-7 Supplemental Information 7 Gene Ontology (GO) annotation of HT-Specific DAPs in response to heat stress

10.7717/peerj.11509/supp-8 Supplemental Information 8 The DAPs involved in mainly metabolic processes in pepper under heat stress

## Additional Information and Declarations

### Data Availability

The following information was supplied regarding data availability: The mass spectrometry proteomics data are available at the ProteomeXchange Consortium via the PRIDE partner repository: PXD019556.
