# Supplementary figures and images for: iTRAQ-based quantitative proteomic analysis of heat stress-induced mechanisms in pepper seedlings
Source: PeerJ. 2021 Jun 3;9:e11509. doi: 10.7717/peerj.11509 (PMC8180192; doi:10.7717/peerj.11509)

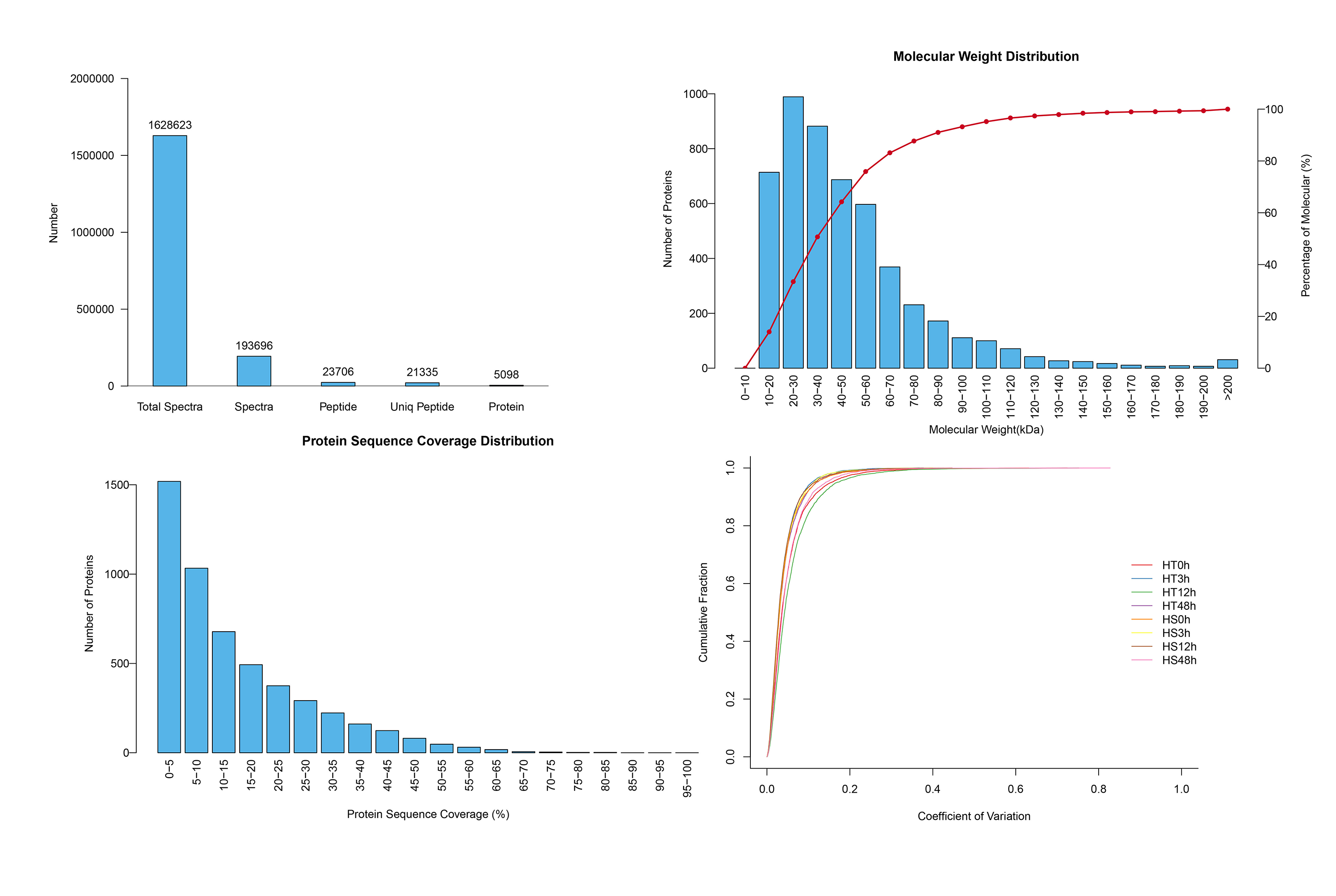

Supplement: Supplemental Information 1 [file peerj-09-11509-s001.png]

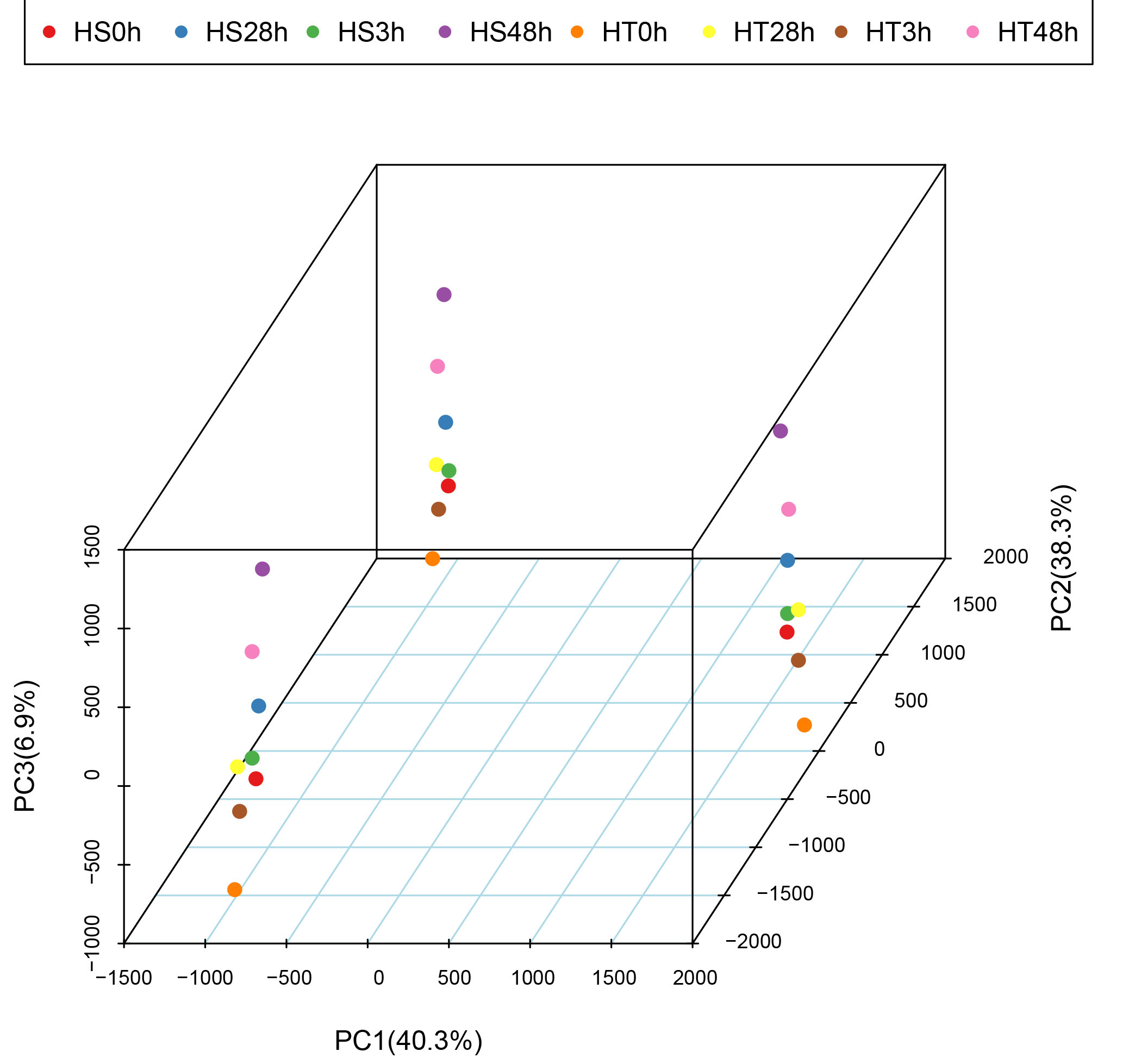

Supplement: Supplemental Information 2 — (A) Statistics of basic information of protein identification, (B) protein mass distribution, (C) distribution of protein sequence coverage, and (D) coefficient of variation of replicates. [file peerj-09-11509-s002.png]
